# Short-time AOIs-based representative scanpath identification and scanpath aggregation

**DOI:** 10.3758/s13428-023-02332-w

**Published:** 2024-01-09

**Authors:** He Huang, Philipp Doebler, Barbara Mertins

**Affiliations:** 1 Department of Statistics, TU Dortmund University, 44227 Dortmund, Germany; 2 Departments of Cultural Studies, TU Dortmund University, 44227 Dortmund, Germany

**Keywords:** Eye-tracking, Scanpaths, Aggregation, Representative scanpath, Scanpath clustering

## Abstract

A new algorithm to identify a representative scanpath in a sample is presented and evaluated with eye-tracking data. According to Gestalt theory, each fixation of the scanpath should be on an area of interest (AOI) of the stimuli. As with existing methods, we first identify the AOIs and then extract the fixations of the representative scanpath from the AOIs. In contrast to existing methods, we propose a new concept of short-time AOI and extract the fixations of representative scanpath from the short-time AOIs. Our method outperforms the existing methods on two publicly available datasets. Our method can be applied to arbitrary visual stimuli, including static stimuli without natural segmentation, as well as dynamic stimuli. Our method also provides a solution for issues caused by the selection of scanpath similarity.

## Introduction

Eye-tracking has been widely used in applied and scientific research over the past decades. It allows researchers to study the movements of a participant’s focus of visual attention during a variety of activities and thus provides insight into the cognitive processes underlying human behaviors (Eckstein et al., 2017; Hartmann & Fischer, 2016; Koć-Januchta et al., 2017; Peterson et al., 2015). Due to the high degree of viewing freedom, one challenge for the researcher is to understand the scanning strategies in a sample. The present paper proposes a novel method to identify a sample-level aggregated scanning strategy using a representative scanpath.

## Scanpaths

**Fig. 1:**
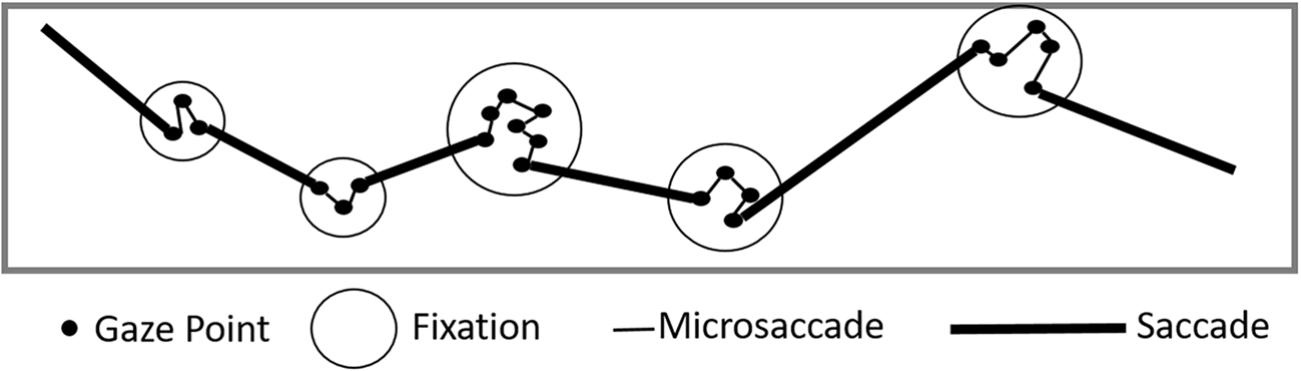
An example of a scanpath

In many applications, the recordings of the point of regard (POR) provided by the eye-tracking equipment are reduced to scanpaths. A scanpath (see Fig. 1) is the temporal sequence of spatial positions of fixations and saccades. Fixations occur when the gaze pauses over informative regions, and saccades are rapid movements between fixations (Salvucci & Goldberg, 2000). The micromovements within the fixation are classified as noisy low-level oculomotor phenomena and are subdivided into microsaccades, tremor and drift, which are involuntarily produced during fixation periods. This reduction is convenient to minimize the complexity of eye-tracking data while retaining its most essential characteristics in higher-level analyses. Scientists have proposed various ways of identifying fixations. Most of them are based on thresholds of velocity, distance or dispersion, duration, and angle of the POR. The choice of method may depend on the specific research question and the nature of the eye-tracking data being analyzed. In this paper, we focus on the resulting scanpaths.

## Areas of interest

**Fig. 2:**
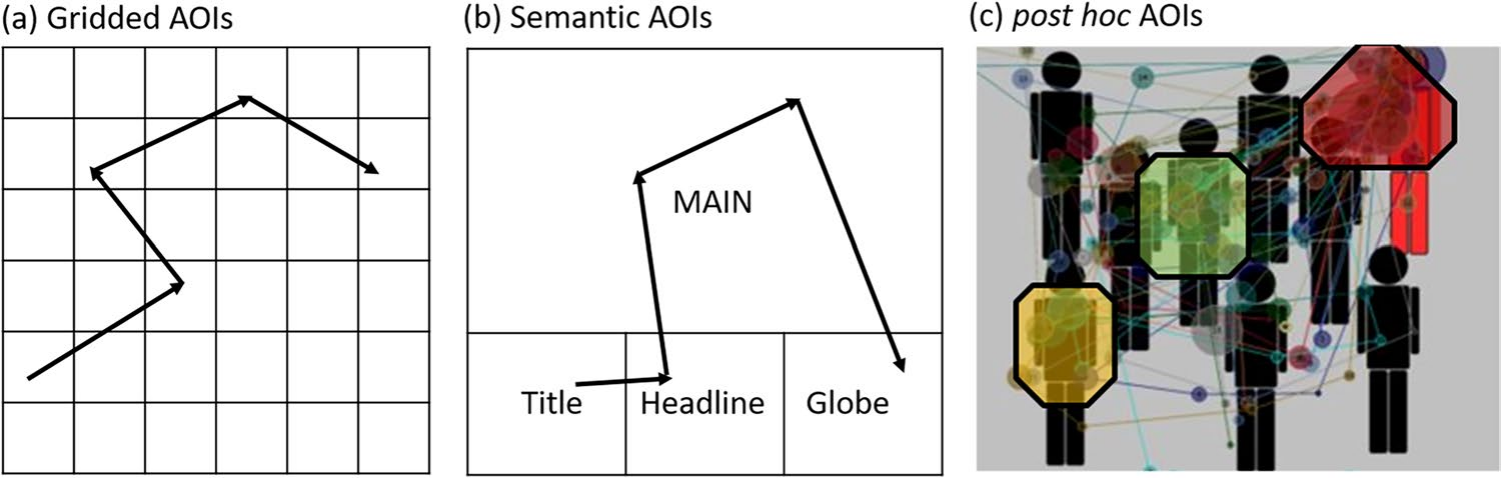
An example of a priori AOIs and post hoc AOIs. Gridded AOIs (**a**), semantic AOIs of a web page (**b**), and post hoc AOIs (**c**)

**Fig. 3:**
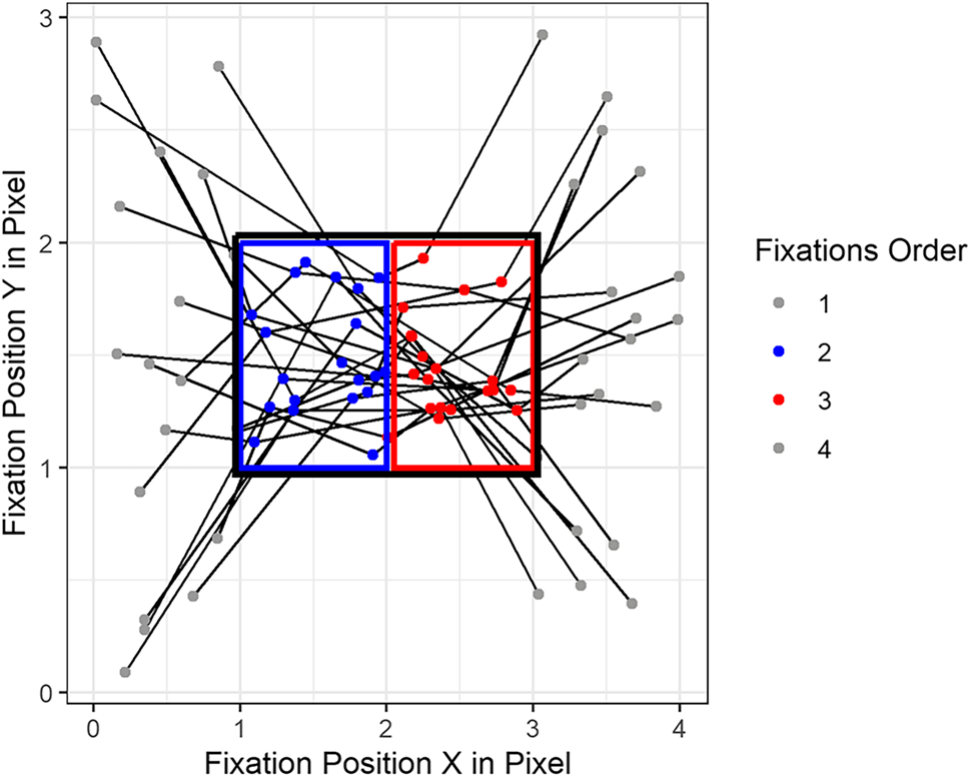
An example of short-time AOIs (*red* and *blue boxes*) and global AOIs (*black box*) obtained from the fixations on a group of random generated scanpaths

As stated in Gestalt theory (Kanizsa, 1979), the elements in bounded areas are perceived as belonging together, so that a single fixation can be located at anywhere of the bounded areas. Therefore, the location of the fixations should be understood in the context of the larger visual scene, namely area of interest (AOIs). As a result, a study of scanpaths should be based on the AOIs that they pass through, or the AOIs estimated from them. It is important to distinguish between a priori AOIs, which can be obtained before viewing and post hoc AOIs, which are derived from the fixations on scanpaths. A priori AOIs can be divided into gridded AOIs and semantic AOIs. Gridded AOIs, as shown in Fig. 2a, are created by placing grids with equal size over the stimulus, which ignores the semantic aspects of the stimulus. Semantic AOIs, as shown in Fig. 2b, are the natural segmentation of the stimulus. Semantic AOIs are used, among other applications, for webpages, since webpages can be automatically segmented into rectangular visual elements (= AOIs) using the underlying source code (Akpınar & Yeşilada, 2013). Post hoc AOIs, as shown in Fig. 2c, are the areas that contain relatively many or long time fixations. The post hoc AOIs formed by the fixations on scanpaths from different observers can be detected by cluster algorithms like *k*-means, DBSCAN, and OPTICS, which group fixations with similar positions (He et al., 2017; Latimer, 1988; Naqshbandi et al., 2016). The advantage of post hoc AOIs are that they can be obtained automatically. Empirically, post hoc AOIs take into account the semantics of the stimuli to some extent. The disadvantages are that fixations are treated as isolated points on the two-dimensional plane. The temporal order and the duration attribute of the fixations are ignored in existing scanpath aggregation approaches. The consequence of this disadvantage can be well explained with the simple example shown in Fig. 3. Twenty scanpaths are randomly generated, so that the second fixations are uniformly distributed in the blue box and the third fixations are uniformly distributed in the red box. However, since the red and blue boxes are so close to each other, the post hoc AOI obtained by a clustering algorithm will most likely be the black box. Such an AOI covers two spatially close but semantically different AOIs, namely the blue box and red box. In particular, when the stimulus is a dynamic video, spatially identical or close but temporally different fixations may come from completely different visual elements. Therefore, the post hoc AOIs obtained from all the fixations fail to describe the dynamic movement pattern of scanpaths accurately. In this paper, we distinguish between short-time AOIs in a limited time segment (e.g., the blue and red boxes) and global AOIs across all time points (e.g., the black box).

## Scanpath aggregation by representative scanpaths

The dynamic movement pattern in a group of scanpaths can be extracted by a representative scanpath, which can be seen as an aggregated scanning strategy for a sample of viewers. A representative scanpath *s*^∗^ can be an existing scanpath in the sample, which minimizes the average distance from other sample members. Formally, given a sample of scanpaths *S* = {*s*^1^, ⋯, *s*^*M*^},

1 $${s}^{\ast }=\arg\ {\min}_{s\in S}\frac{1}{M}\sum_{i=1}^Md\left(s,{s}^m\right),$$

where *d*(·, ·) is the scanpath dissimilarity. For example, von der Malsburg and Vasishth (2013), Parshina et al. (2022), and Paape et al. (2022) cluster the scanpaths into several groups and find for each group a scanpath that minimizes the average Scasim (von der Malsburg & Vasishth, 2011) dissimilarity as the prototypical view pattern for that group.

Instead of identifying an existing representative scanpath from the sample, some methods have been proposed to generate an artificial scanpath to represent the group. Their common approach is to first define or find AOIs and then derive a representative scanpath from the AOIs (see Table 1). Eraslan et al. (2014) represent the scanpath with a string by replacing the fixations on it with a shorthand name of the semantic AOI. For example, the scanpath on the middle panel in Fig. 2 will be represented T - H - M - M - G (where T stands for ‘Title’ etc.). Then, a common subsequence shared by all subjects is identified as the representative scanpath. This method is limited to the cases where no common subsequence shared by individuals exists. It can be improved by the method of Hejmady and Narayanan (2012), who instead find the frequent subsequence supported by a specified number of subjects with the sequential pattern mining algorithm (Ayres et al., 2002). Similarly, Wang et al. (2022) detect frequent sub sequence using the *k*-mer analysis, which counts the frequency of subsequence of neighbouring elements of length *k* (Manekar & Sathe, 2018). The common limitation of these semantic-AOIs-based methods is that they are only suitable for visual stimuli where natural segmentation exists or that can be easily segmented by a computer, such as webpages. However, for an arbitrary image, it is usually difficult to make a decision on how to segment it. Such segmentation is usually subjective, and different ways of segmentation lead to different strings for scanpaths. The method of scanpath aggregation based on post hoc AOIs can well avoid the problems caused by segmentation and is applicable to stimuli without existing segmentation. The heuristic method and the Candidate-constrained Dynamic Time Waring Barycenter Averaging (CDBA) method proposed by Li and Chen (2018) obtained the segmentation of stimuli through post hoc AOIs by clustering the fixations on scanpaths. Their methods try to search a scanpath *s*^∗^ that minimizes the average distance to all the scanpaths in sample, as shown in Eq. (1). However, the search space is no longer limited to the original sample *S*, but a set $\overset{\sim }{S}$ of reconstructed scanpaths. Scanpaths in $\overset{\sim }{S}$ are constructed by connecting the centers of the post hoc AOIs. Since the AOI centers can be used repeatedly in the same scanpath, the set $\overset{\sim }{S}$ can contain a huge number of reconstructed scanpaths. To reduce the computational effort, the length of the scanpaths in $\overset{\sim }{S}$ (number of fixations) is limited to the maximum length of the original scanpaths. Even so, the set $\overset{\sim }{S}$ is still large and traversing all the scanpaths in it, namely the heuristic method, is very time consuming. A compromise is to initialize a random scanpath and then update the scanpath according to certain rules iteratively, namely the CDBA method, which achieves a local optimum after a smaller number of updates. Heuristic and CDBA methods can be used to automatically find a representative scanpath in a sample of scanpaths for any static visual stimuli. However, the fixations on the detected representative scanpath are the centers of the post hoc AOIs obtained from all the fixations without considering their order and time period, namely the global AOIs; cf. the black box shown in Fig. 3. Such fixations fail to represent the location of the fixations in a particular time period. A better way is to use the short-time AOIs; cf. the blue and red boxes in Fig. 3.

**Table 1:** Methods of AOI-based scanpath aggregation

Method	AOI	Description
Eraslan et al. (2014)	A priori (semantic)	(i) Represents scanpaths as string; (ii) find common string shared by all scanpaths
Hejmady & Narayanan (2012)	A priori (semantic)	(i) Represents scanpaths as string; (ii) find frequent strings shared by a majority of scanpaths
Wang et al. (2022)	A priori (semantic)	(i) Represents scanpaths as string; (ii) detect frequent sub sequence using the *k*-mer analysis
CDBA & Heuristik (Li & Chen, 2018)	Post hoc (global)	(i) Construct a candidate set of representative scanpaths; (ii) find an optimal scanpath in the candidate set that minimizes the average DTW distance to the original scanpaths

In addition to the above methods, Burch et al. (2019) developed a visualization tool to aggregate the scanpaths. They first identify the post hoc AOIs based on the density of the fixations on the stimulus, and then present a hierarchical flow chart between the AOIs, in which the thickness of the lines between AOIs is determined by the transfer probability between them. This tool does not actually generate a representative scanpath, but visually aggregates the scanpaths hierarchically, which can also help us to understand the view patterns of a group. However, due to using of the post hoc AOIs, this method has the same limitation as Li and Chen (2018).

In this paper, we propose a new algorithm to identify short-time AOIs from a group of scanpaths and get the representative scanpath by linking the centers of the short-time AOIs. The basic idea to obtain short-time AOIs is to cluster the fixations from different time periods separately. The definition of short-time AOIs and how to detect them automatically will be explained formally and in detail in next section. Then, we evaluate our method on two publicly available eye-tracking data sets. Results show that the generated scanpath can be more representative than any existing scanpath from the sample.

## Methodology

In this section, we first present an overview of our design, including a formal problem statement and the skeleton of our algorithm, which we call Short-Time-AOI Scanpath Aggregation (STASA). After that, we describe each step of our method in detail.

## Problem statement

**Fig. 4:**
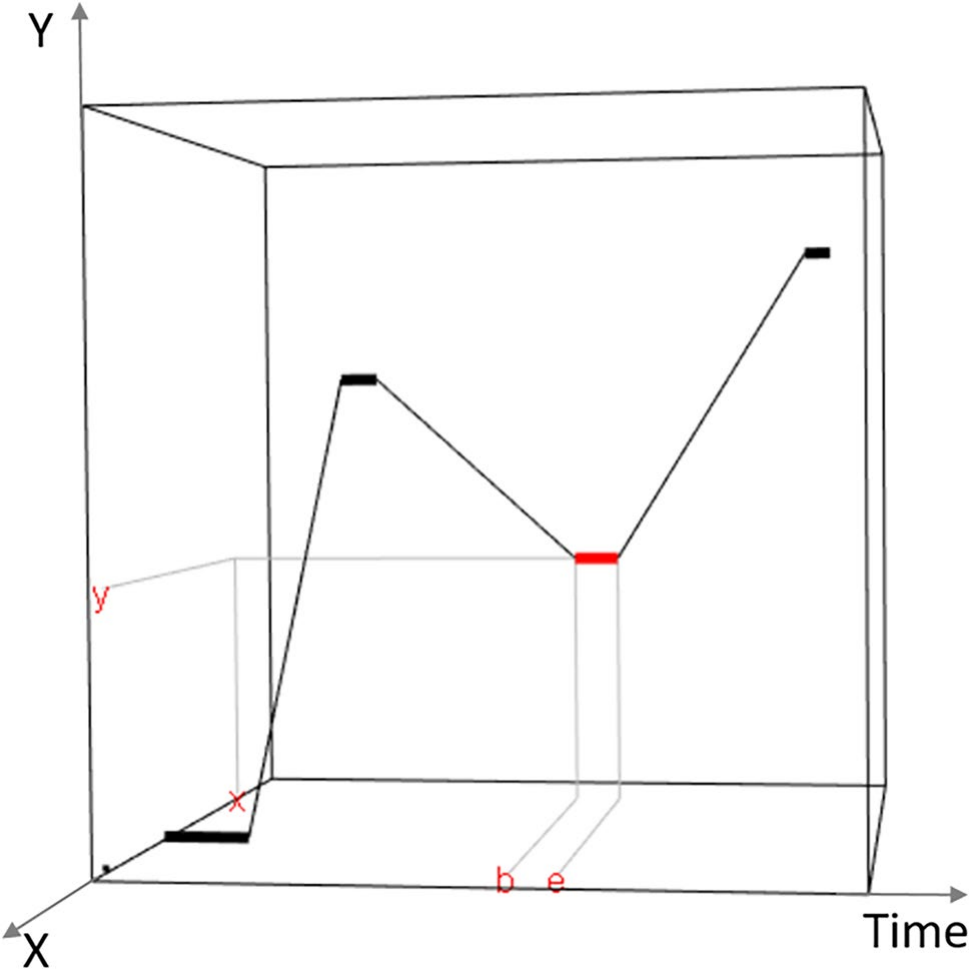
An example of scanpath with four fixations in 3D. The third fixation (in *red*) locates on (*x*, *y*) and last from *b* to *e*

A fixation on the two-dimensional (2D) plane can be denoted by a quadruple (*x*, *y*, *b*, *e*), in which (*x*, *y*) is the space location of the fixation in pixel coordinates and (*b*, *e*) with *b* < *e* are the start time point and end time point of the fixation. Thus, a scanpath *s* with *n* fixations is a sequence of fixations with *s* = {(*x*_1_, *y*_1_, *b*_1_, *e*_1_), ⋯, (*x*_*n*_, *y*_*n*_, *b*_*n*_, *e*_*n*_)} and *b*_1_ < *e*_1_ < *b*_2_ < *e*_2_ < ⋯ < *b*_*n*_ < *e*_*n*_. Figure 4 shows an example of a scanpath with *n* = 4 in a three-dimensional (3D) coordinate system resulting from adding a time axis. Given a set of scanpaths {*s*^1^, ⋯, *s*^*M*^}, with ${s}^m=\left\{\left({x}_1^m,{y}_1^m,{b}_1^m,{e}_1^m\right),\cdots, \left({x}_{n_m}^m,{y}_{n_m}^m,{b}_{n_m}^m,{e}_{n_m}^m\right)\right\}$, for *m* = 1, ⋯, *M*, we develop an efficient algorithm to generate a representative scanpath *s*^∗^. Following Li and Chen (2018), the representativeness of *s*^∗^ can be evaluated by its average distance to all scanpaths in the sample

2 $$\frac{1}{M}\sum_{i=1}^Md\left({s}^{\ast },{s}^m\right).$$

In contrast to Li and Chen (2018), we do not fix *d*(·, ·) as the Dynamic Time Warp Distance (Vintsyuk, 1968) because it can only measure the distance of two scanpaths by considering them as trajectories, which ignores the difference of duration of the fixations on them. Anderson et al. (2015) provide an overview of common scanpath similarity measures. As dissimilarity measures can always be transformed to similarity measures, we use this two concepts interchangeably in this paper. These similarity measures fall into two categories. One focuses on only one or two aspects of scanpath similarity, such as fixation overlap and gaze shift of two scanpaths (Shepherd et al., 2010), linear distance of fixations (Mannan et al., 1995; Mathôt et al., 2012), and cross-recurrence (Anderson et al., 2013). Therefore, we do not use them in this paper. Another category of similarity measures covers as many aspects of scanpath similarity as possible, i.e., spatial and temporal similarity of fixations and order of fixations. They are the Levenshtein distance (Levenshtein et al., 1966), the Scasim (Von der Malsburg & Vasishth, 2011), ScanMatch (Cristino et al., 2010), and MultiMatch (Jarodzka et al., 2010). A brief description of these methods follows, as they are used in this paper to compute the average similarity in Eq. (1) and evaluate the representativeness of the obtained scanpath.

Levenshtein distance measures the difference between two strings, so that the scanpath has to be transformed into strings of AOIs first. The distance between two strings is the minimum number of single character edits (insertions, deletions, or substitutions) required to transform one string into the other.

Scasim is a dissimilarity measure based on the Levenshtein distance. Instead of converting the scanpath into a string, it generalizes the character editing operations to penalize the distance between fixations taking into account the high acuity in the fovea and the drop in resolution towards the periphery.

ScanMatch first converts scanpaths into strings after placing grids with equal size on the stimulus (see Fig. 2a) and names the grids with letters. A letter is repeated a certain number of times depending on the duration of the fixation on the corresponding grid cells, e.g., each individual letter represents a 50-ms bin. The resulting string sequence incorporates spatial location, sequential information, and temporal durations of the fixations on the scanpaths. Therefore, comparing them provides an overall similarity of the scanpaths. The strings are aligned and compared using Needleman and Wunsch (1970) algorithm. The Needleman–Wunsch algorithm creates a matrix with all scoring possibilities based on a substitution matrix, which provides a score for aligning two AOI letters. The substitution matrix encode information about the relation between each AOI (grid). The relationship can be based on the semantic segmentation of an image, or basically the Euclidean distance between the grids. Therefore, the similarity calculated by ScanMatch depends highly on the number of grids and the substitution matrix used in Needleman–Wunsch algorithm.

MultiMatch represents the two scanpaths to be compared as sequences of saccade vectors (the vectors between each two temporally consecutive fixations), say $\left({\overrightarrow{u}}_1,\cdots, {\overrightarrow{u}}_m\right)$ for one scanpath and $\left({\overrightarrow{v}}_1,\cdots, {\overrightarrow{v}}_n\right)$ for another. The saccade vectors on two scanpaths are aligned and compared from five perspectives, i.e., vector similarity, direction similarity, length similarity, position similarity, and duration similarity. The vector similarity is the average difference of the aligned saccade vector pairs, namely ||$\overrightarrow{u}-\overrightarrow{v}$||, which actually incorporates the direction similarity (angle between $\overrightarrow{u}$ and $\overrightarrow{v}$) and length similarity (||$\overrightarrow{u}$|| - ||$\overrightarrow{v}$||). The position similarity and duration similarity are for the fixations on the two ends of the saccade vectors. Position similarity is linked to their spatial distance, and duration similarity is their difference in duration.

In summary, Scasim calculates scanpath dissimilarity directly based on the pixel coordinates of the fixation, whereas ScanMatch replaces the fixations with the letters of grids they are located and calculates the similarity based on the grid’s distance. The saccade vector used by MultiMatch is also based on the pixel coordinates of the fixation, but MultiMatch simplifies scanpaths firstly based on angle and amplitude of the saccades before calculating their similarity. After simplification, spatially closed fixations are merged. In this way, MultiMatch is similar to ScanMatch, where spatially closed fixations are also most likely be merged by the grids, depending on the size of the grids. In addition, ScanMatch and Scasim provide a single score for the overall similarity of two scanpaths, whereas MultiMatch provides five scores for each of the five different aspects.

## Overall procedure of STASA

**Fig. 5:**
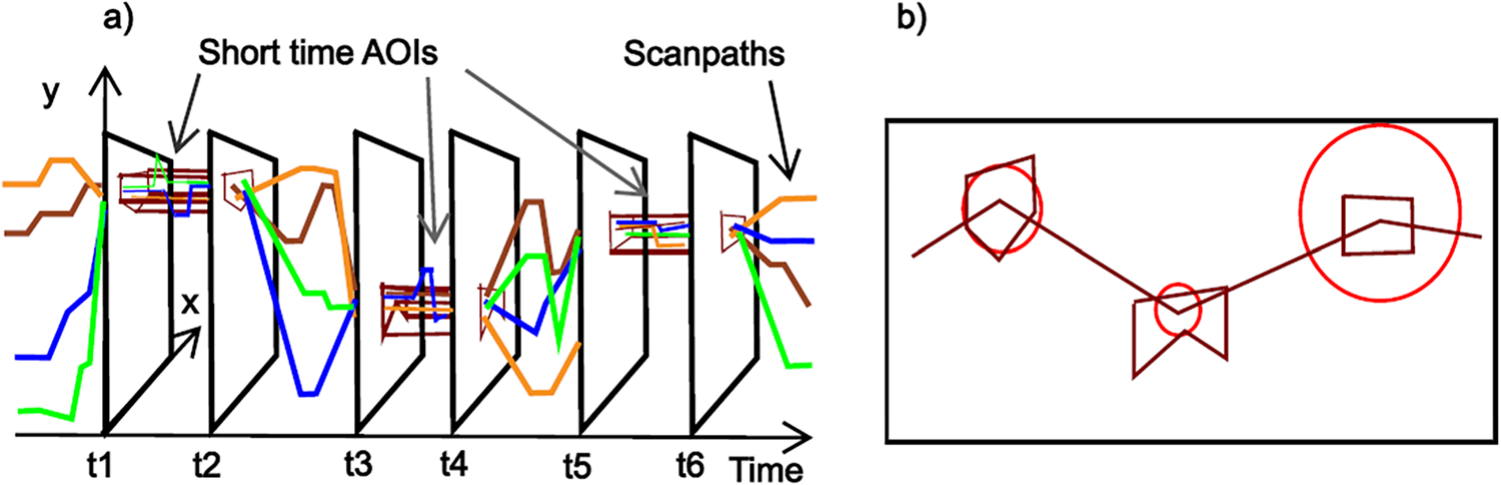
From short-time AOIs to representative scanpath. **a** Scanpaths and short-time AOIs in 3D. **b** Short-time AOIs and representative scanpath in 2D

The principal idea of STASA is to get short-time AOIs first and then obtain “synthetic” fixations of the representative scanpath from short-time AOIs. In Fig. 5a we display the scanpaths in a three-dimensional (3D) coordinate system after adding a time axis. The four scanpaths in this figure concentrate more or less in the upper part of the stimulus from time point *t*_1_ to *t*_2_, and concentrate in the middle part of the stimulus from time point *t*_3_ to *t*_4_, and so on. These areas of concentration are short-time AOIs. A short-time AOI lasts from one time point to another, and has a bounded range in space, which can be denoted as (*P*, *b*, *e*), where *P* the set of vertices of the bounding polygon, and (*b*, *e*) are the start and end time point. After we obtain the short-time AOIs, we link them (and the fixations derived from them) in time order to get a representative scan path for the scanpaths. As shown in Fig. 5b, we display the representative scanpath together with its short-time AOIs back on the two-dimensional (2D) stimulus, which is a natural way of visualization for static stimuli. The polygons indicate the shape and location of the short-time AOIs and the area of the cycle is proportional to the duration of the short-time AOIs.

**Fig. 6:**
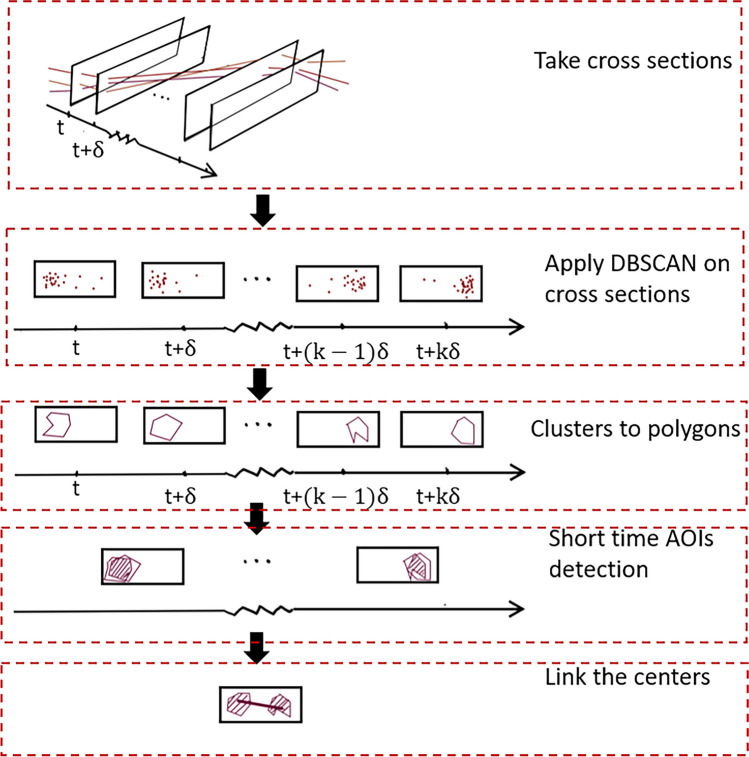
Overall procedure of STASA

As shown in Fig. 6, our method consists of five steps: i) Represent scanpaths in a 3D coordinate system and take cross-section slices along the time axis; ii) Apply DBSCAN (Ester et al., 1996) clustering algorithm on the fixations on each slice; iii) Surround each cluster of fixations on each slice by a polygon; iv) Determine short-time AOIs based on the similarity of polygons on adjacent slices; v) Determine the fixations of the representative scanpath from short-time AOIs. Each of these steps is explained in detail in the following subsections.

## Take cross section slices along the time axis

All scanpaths are start-time aligned and represented in the 3D coordinate system. We take cross-section slices along the time axis with a small equal time distance *δ*, and get the space locations (*x*, *y*) of all fixations from all scanpaths on each slice. A smaller *δ* makes the calculation more accurate but increases the computational effort. For smaller data sets (usually a few dozen scanpaths with a few seconds of experimentation time), we can use the sampling interval of the original eye-tracking data, which is the inverse of the sampling frequency of the eye-tracker used. For dynamic stimuli, a slice can be a single frame of the video.

## Apply DBSCAN on slices

**Fig. 7:**
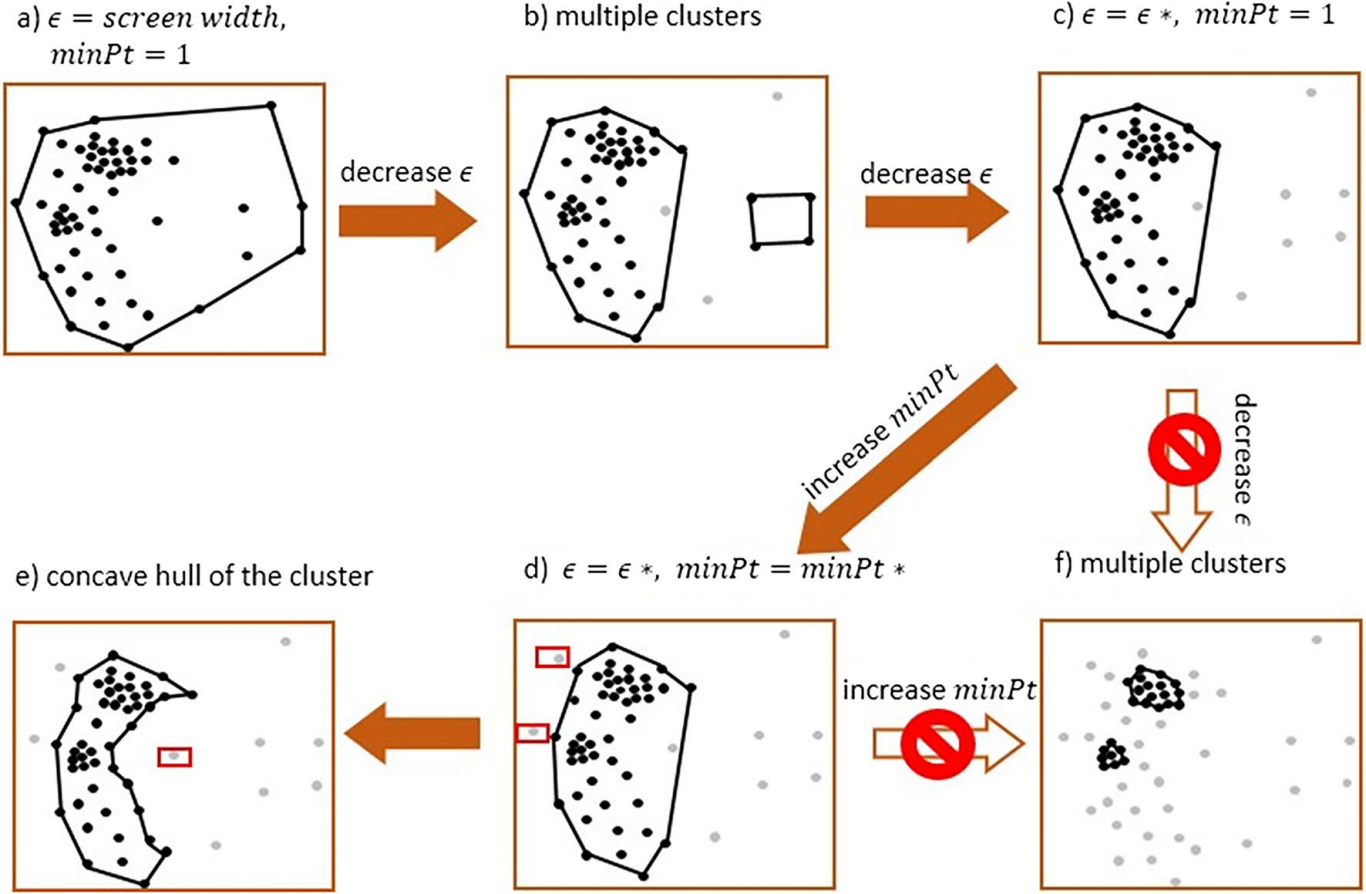
The process of determining the relatively large and dense cluster for each cross-section slice and obtaining the concave boundary of the cluster

For fixation points on each cross-section slice, we use the DBSCAN (Ester et al., 1996) clustering algorithm to remove outliers and find relatively dense areas on that slice. The principal idea of DBSCAN is to find dense areas and form clusters by expanding the dense areas recursively. The number of clusters is not prespecified and is determined by DBSCAN. The dense area is formed by those points that have at least *minPt* neighboring points within a given search radius *ϵ*. Compared with other traditional clustering algorithms like *k*-means, DBSCAN is well suited for detecting AOIs on visual stimuli, since it detects arbitrarily shaped clusters without apriori knowledge of the stimuli, whereas *k*-means and other clustering algorithm often prefer roughly circular AOIs. The choice of parameters (*minPt*, *ϵ*) is crucial to our algorithm. Different parameter values will result in different numbers and sizes of clusters. Because we will later compare the similarity of clusters on two adjacent slices, having multiple clusters on the same slice will make the later steps difficult. Since our goal is to find a common-view pattern for a group of scanpaths, we identify only one major cluster for each slice. This cluster should i) include as much points as possible; ii) include points as dense as possible. The density of the cluster can be controlled by the parameter *ϵ*; the smaller the *ϵ*, the higher the density, and the minimum number of points in the cluster is restricted by *minPt*. To achieve our goal, we get the optimal (*ϵ*^∗^, *minPt*^∗^) as follows:

- For each slice by applying DBSCAN, we start with a large *ϵ* (width of the visual stimulus) and a small *minPt* (half of the number of scanpaths in set *L*), which will result in one cluster containing all the points as shown in Fig. 7a.
- Then we keep *minPt* fixed and decrease *ϵ* by one pixel gradually. The reduction of *ϵ* requires denser points within the cluster, which causes the original cluster formed under larger *ϵ* to split into two or more denser clusters and some isolated points (the grey points in Fig. 7b) are no longer contained within any cluster.
- Then we continue to reduce *ϵ*, so that more isolated points are excluded from the clusters and the relatively sparse clusters disappear. We stop decreasing *ϵ* when only the last densest cluster is left (see Fig. 7c). This *ϵ* value is the searched optimal value *ϵ*^∗^.
- Next, we keep the value of *ϵ* fixed by *ϵ*^∗^, and increasing the value of *minPt* by one gradually. An increase in *minPt* also requires denser points within the cluster, which excludes some distant points at the margin of the cluster, as shown in the red borders in Fig. 7d. However, it may also lead the cluster to split into multiple clusters. We choose the maximal value of *minPt* so that the number of clusters remains at one as the searched value *minPt*^∗^.

## Surround clusters with polygons

The cluster obtained by DBSCAN on each slice is a collection of points. To estimate a boundary of the cluster, we obtain the concave hull, which is a polygon using the approach of Park and Oh (2012). A concave hull is a shrink-wrapping of a set of points, which has a smaller area compared to the convex hull and represents a natural boundary of the points. From the simple example shown in Fig. 7d, e, we can see that the concave hull is better than the convex hull to reflect the geometrical characteristics of fixations. The outlier points in the red box are now no longer in the cluster.

## Short-time AOIs determination

After we obtain the concave polygons on each slice, we determine the short-time AOIs. First, we determine the time interval (*b*, *e*) of the short-time AOIs as follows.

**Fig. 8:**
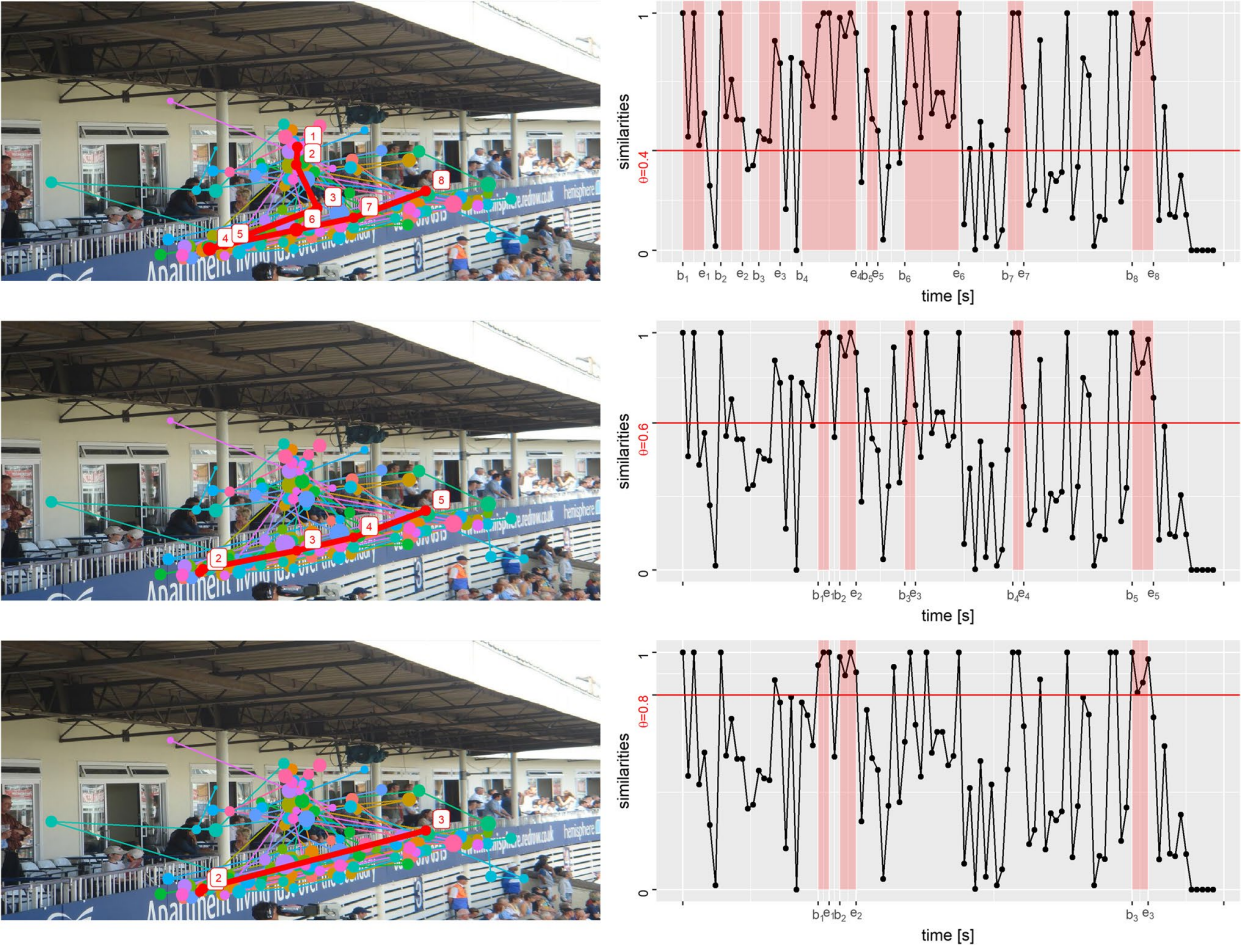
An example of representative scanpaths with different values of *θ*. The scanpaths are in the left panels. The *thick red line* is the representative scanpath, the *other colored lines* are scanpaths of individual observers. The fixations on representative scanpaths are numbered by time order. The *right panels* show how the time intervals of the short tine AOIs are obtained under corresponding *θ*

We calculate the similarity *S*_(*i*, *i* + 1)_ of the polygons on *i*-th and (*i* + 1)-th slice for all *i* with

3 $${S}_{\left(i,i+1\right)}=\frac{I_{\left(i,i+1\right)}}{U_{\left(i,i+1\right)}},$$

where *U*_(*i*, *i* + 1)_ is the combined area of the polygons on slice *i* and *i* + 1, and *I*_(*i*, *i* + 1)_ the overlapped area of the polygons on slice *i* and *i* + 1. The similarities *S*_(*i*, *i* + 1)_ against the time point *i* ∗ *δ* can be displayed in a scatter plot as shown in the right panel of Fig. 8. The time intervals (*b*_*j*_, *e*_*j*_) of the short-time AOIs are those in which all the calculated similarities are greater than a predefined threshold *θ*. Extremely short intervals, which contain less than three slices, are excluded, making sure that the duration of the resulting fixation is no shorter than 100 ms. We can see that under different *θ* we obtained different representative scanpaths. How to choose the threshold *θ* will be discussed latter.

After we obtained the time intervals of the short-time AOIs, we compute the spatial scope of them as the union of areas surrounded by the polygons on all the slices within the corresponding time interval. Denote by *p*_*i*_ the obtained polygon on *i*-th slice, then the polygon *P*_*j*_ of *j*-th short-time AOIs is defined as

4 $${P}_j=\bigcup_{\delta \cdot i\in \left[{b}_j,{e}_j\right]}{p}_i$$

Suppose we get *J* short-time AOIs in the previous step. Then the representative scanpath *s*^∗^ would consist of *J* fixations. The spatial location of *j*-th fixation (*x*_*j*_, *y*_*j*_) is the geometric center of the polygon *P*_*j*_, and the start time and end time of *j*-th fixation is the start time and end time of the *j*-th short-time AOI, namely *b*_*j*_ and *e*_*j*_, for all *j* ∈ {1, ⋯, *J*}. The advantage of using the geometric center is that it has the smallest distance to the vertices of the AOI. However, if we get a short-time AOI that is very close to the edge of a circle, then its geometric center will be surrounded by this AOI, but it will not be on part of this AOI. It is also difficult to use any point on the circle to represent the position of a circle. However, this is not due to the use of the concave hull, since the geometric center of the concave hull and convex hull in this case are the same or similar. The reason we chose concave hull is to make the resulting AOI as much as possible in the region of dense fixations. Therefore, when visualizing the scanpath, we recommend displaying the scanpath together with the short-time AOIs in which each fixation is located, as shown in the simplified diagram in Fig. 5b. The visualization function of our package also provides the option for users to show the AOIs together with the scanpath.

## Choice of the threshold parameter

The threshold *θ* we mentioned above is a tuning parameter of our method. A smaller *θ* will combine polygons on two adjacent slices, say *i*-th and (*i* + 1)-th slices, with smaller overlapping area to form a larger short-time AOIs. There are two possible reasons for the small overlap of polygons on adjacent slices. i) The scanpaths are temporally highly unsynchronized. For example, a group of scanpaths (say each with *n* fixations) are spatially almost identical (focusing on the same visual elements), but the duration of each fixation is different, so that they are not synchronous. Then our task is to generate a scanpath that is also spatially almost identical to these scanpaths, but where the duration of each fixation is the overlap time of the *n* fixations on all the scanpaths at the similar location. The parameter *θ* can be used deal with this situation. *θ* is the minimum requirement for the similarity of AOIs on two adjacent cross sections within the same short-time AOI. If the scanpaths are perfectly synchronized, then the resulting AOIs on each cross section will be identical within the same short-time AOI, requiring the value of *θ* to be set to one in order to distinguish between different short-time AOIs (fixations at different locations). However, in most cases, observers are not synchronized due to the different durations of each fixation, even though their scanpaths are spatially similar. We assume that their fixations on similar locations overlap in time. Then different AOIs will be formed on different cross sections, as different cross sections contain different points from these *n* fixations. In such a case, different values of *θ* tolerate different degrees of degree of desynchronization. ii) The two polygons are from two different visual elements. Most observers’ fixations were on one of the visual elements at the time point corresponding to the *i*-th slice and on the other visual element at the time point of (*i* + 1)-th slice. However, again due to the high degree of view freedom, a few observers gazed earlier on another visual element at the time point of the *i*-th slice or stayed on the previous visual element at the time point of (*i* + 1)-th slice. This results in two polygons both covering two different visual elements. In this case, a larger *θ* can result in two spatially different AOIs, corresponding to two different visual elements of the stimulus, instead of their combination.

Figure 8 shows three different representative scanpaths obtained by our method with three different values of *θ*. The left panels contain representative scanpaths and right panels illustrate how the time interval of the short-time AOIs are obtained under the corresponding value of *θ*. The image is one of the 1003 stimuli in the MIT1003 data set (Judd et al., 2009), which was observed by 15 individuals. For convenience, we only show three cases with *θ* at 0.4, 0.6, and 0.8 from top to bottom. All the three representative scanpaths contain the horizontal path at the bottom of the stimulus, which is the most common view pattern of the eye movement in this sample. As *θ* increases, the representative scanpath tends to be shorter and contains no longer the vertical eye movement at the beginning of the viewing. This is because at the beginning of the experiment, the gaze of the viewer is attracted to the center of the screen due to a centered fixation cross before the stimulus is displayed. After the stimulus is displayed, the viewers move their gaze at different speeds. Some viewers were quicker to target the texts on the wall and then moved their gaze to the right horizontally. Hence, fixations at the very beginning of the viewing cannot form short-time AOIs under larger *θ* because they are actually far apart in time.

The choice of *θ* also depends on how the representativeness of the scanpath is defined. A suitable definition is usually multidimensional, such as the five dimensions of MultiMatch. In practical applications, we recommend tuning *θ* between 0.1 and 0.9 spaced by 0.1, and then select the *θ* that minimizes the average dissimilarity to all the scanpaths in sample. The dissimlarity is recommended to be Scasim. Scasim is chosen for the following reasons. As previously described, most scanpath similarities focus on only one aspect of the scanpath similarity, while Levenstein distance, Scasim, ScanMatch, and MultiMatch are able to cover both spatial and temporal aspects of the scanpath similarity. MultiMatch, however, gives five scores for five different aspects of scanpath. Making one score larger would reduce some other scores. Levenstein distance and ScanMatch are also excluded as the default because they have to divide the stimuli in advance into different regions (or gids). Different divisions will lead to different results. In addition, we only tried nine different theta values between 0.1 and 0.9. The user could also try more values, but based on experiments on two large datasets, we do not think it is necessary, since the difference of the scanpaths obtained by the neighboring *θ*’s values is not significant.

Furthermore, if the scanpaths within the samples are very different from each other, we may obtain an unrealistic representative scanpath with extremely short fixation durations or extremely long saccade durations, as shown in the right panel of Fig. 8 by *θ* = 0.8. In this case, we should choose a smaller *θ* to avoid such an unrealistic scanpath. However, the user does not have to choose the value of *θ* manually. Different *θ* values will generate scanpaths with different representativeness (i.e., average similarity with all the scanpaths of the sample). By default, our method will automatically select the *θ* that generates a scanpath with the highest representativeness, and an extremely unrealistic scanpath is certainly not highly representative (because it is so different from the real scanpaths). Therefore, the final scanpath will not be the case where *θ* = 0.8, rather more likely the case where *θ* = 0.4. It is possible that the obtained scanpath at the optimal *θ* is still unrealistic, or partially unrealistic. For example, in Fig. 8, at *θ* of 0.4, the time intervals between the fixations (i.e., the saccade duration) of the second half of the scanpath are still very long. If 0.4 is the optimal *θ* value (it may not be, we just show only three values of *θ* for the sake of convenience), then it suggests that these observers have a common observation pattern in the first half of the scanpath, but the second half of the scanpath does not.

## Empirical evaluation

To evaluate our method, we compared the performance of our method with CDBA and heuristic methods on two large public benchmark eye-tracking data sets, namely MIT1003 data set (Judd et al., 2009) and OSIE data set (Xu et al., 2014). The MIT1003 data set contains 1003 natural indoor and outdoor scenes freely viewed by 15 observers for 3 s. The longest dimension of each stimulus is 1024 pixels, and the other dimension ranges from 405 to 1024 pixels.

The OSIE data set contains 700 images freely viewed by 15 observers for 3 s. All the images are of the size 800 × 600 pixels. One difficulty with this data set for us is that it contains only the duration information for the fixations and their exact start and end time points are unknown. That is, the data structure of each fixation in this data set is (*x*, *y*, *d*) instead of (*x*, *y*, *b*, *e*), where *d* is the duration of the fixation. However, our method requires the exact start and end time points to align the scanpaths and to take slices along the timeline. To solve this problem, we constructed artificial start and end time points for each fixation as follows. For each scanpath *s* = {(*x*_1_, *y*_1_, *d*_1_), ⋯, (*x*_*n*_, *y*_*n*_, *d*_*n*_)} in the OSIE data set, we calculated its total saccade time (*ts*) with

5 $$ts=3-\sum_{i=1}^n{d}_i,$$

which is the difference of the total observation time and the total fixation time. We then divide *ts* evenly between each of the two fixations. That is, the saccade time between each two fixations will be set as *ts*/*n*. Thus the start time point of *i*-th fixation can be computed with

6 $${b}_i=\sum_{h=1}^{i-1}{d}_h+\left(i-1\right)\cdot ts/n,$$

and the end time point of *i*-th fixation can be computed with

7 $${e}_i=\sum_{h=1}^i{d}_h+\left(i-1\right)\cdot ts/n.$$

This operation may introduce an error. A more accurate way would be to assign the duration of the saccade based on its amplitudes. However, since the time of saccades only takes up a small percentage of the overall scanpath (in the OSIE data set it is 17% in average), the difference between the artificial start and end time points and their unknown true values will also be small. To simplify the calculations, we divide the saccade time equally as shown in Eqs. (6) and (7). Experiments show that our method still outperforms other methods when this error is included.

Since there exist no ground truth representative scanpaths, for each stimulus the average similarity (or distance) of the obtained representative scanpath to all the 15 scanpaths is used to evaluate the representativeness for that stimulus. As mentioned above, Scasim, MultiMatch, and ScanMatch are used to measure the similarity of two scanpaths. MultiMatch provides five scores for the five dimensions, i.e., vector similarity, direction similarity, length similarity, position similarity, and duration similarity. ScanMatch and Scasim provide a score for the overall scanpath similarity. MuitiMatch and ScanMatch normalize the obtained scores into the interval [0, 1], and the higher the score, the higher the similarity, while Scasim provides score for the dissimilarity in milliseconds, thus the lower the score, the higher the similarity.

For a fair comparison with the CDBA and the heuristic methods, we set the involved parameters of the ScanMatch method to the same as Li and Chen (2018), that is Xbin = 24, Ybin = 18, Threshold = 3.5, GapValue = 0, TempBin = 100. The parameter *δ* of our method are set to 30 ms. *θ* is tuned from 0.1 to 0.9 with distance 0.1.

In Table 2, we present the average representativeness scores for both the MIT1003 and OSIE datasets, each comprising 1003 and 700 stimuli, respectively. The scores are provided for our method, the heuristic method, and the CDBA method. Since the authors of the latter two methods did not publish their source code, the scores of these two methods in this table are from Li and Chen (2018). As shown in Table 2, the average overall representativeness of our method (the scores by ScanMatch) clearly outperform the CDBA and heuristic methods on both data sets. With the five scores of MultiMatch we can identify exactly where this difference comes from. First, the representative scanpaths obtained by our method are on average more representative in terms of the overall vector similarity in both data set, which mainly comes from the high similarity in vector length. The position of the fixations on our representative scanpath is also more representative on both data sets. The position of the fixations in our method are the centers of the short-time AOIs, while in the other two methods, they are the centers of the global AOIs. This is empirical evidence for our key assumptions, i.e., using the centers of the short-time AOIs as the position of fixations can be more representative for the fixations in a particular time period. For duration similarity, our method shows to be advantageous on the OSIE data set, but performs worse on the MIT1003 data set. The CDBA and heuristic methods use the average duration of all fixations within each global AOI as the duration of the fixations for representative scanpath. If we calculate the durations of the fixations in the same way, the score of duration similarity would also be increased (from 0.53 to 0.60 by dataset MIT1003). The disadvantage of using average duration, however, is that it is not possible to give the exact start and end time of each fixation for the representative scanpath, and it may make the total duration of all fixations greater than the actual duration of the viewing, when the number of fixations is large. Therefore, we prefer to calculate the duration of fixations as the difference of the end and start time points of short-time AOIs.

**Table 2:** Average representativeness of different methods on MIT1003 and OSIE data set. The scores are normalized to range from 0 to 1

Dataset	Algorithm	Vector	Direction	Length	Position	Duration	ScanMatch
MIT1003	STASA	0.938	0.736	0.916	0.898	0.553	0.686
	Heuristic	0.882	0.749	0.905	0.874	0.674	0.474
	CDBA	0.882	0.749	0.905	0.875	0.674	0.476
OSIE	STASA	0.948	0.710	0.934	0.885	0.644	0.632
	Heuristic	0.843	0.702	0.850	0.821	0.620	0.415
	CDBA	0.843	0.849	0.820	0.701	0.617	0.416

*Note.* The scores of heuristic and CDBA are from Li and Chen (2018)

Then we compare our method (STASA) with a simple method of finding a scanpath in the raw sample. That is, given the scanpath dissimilarity (or similarity), find a scanpath in the sample that has the smallest average dissimilarity (or largest average similarity) with other sample members. As scanpath dissimilarity and similarity, we choose Scasim and ScanMatch. We denote these two methods as minScasim and maxScanMatch, respectively. For ScanMatch, we divide all stimuli into 12 x 8 grids, the same as the authors of ScanMatch did in their original paper. For STASA, δ of our method are set to 30 ms as default, *θ* is tuned from 0.1 to 0.9 with distance 0.1.

**Fig. 9:**
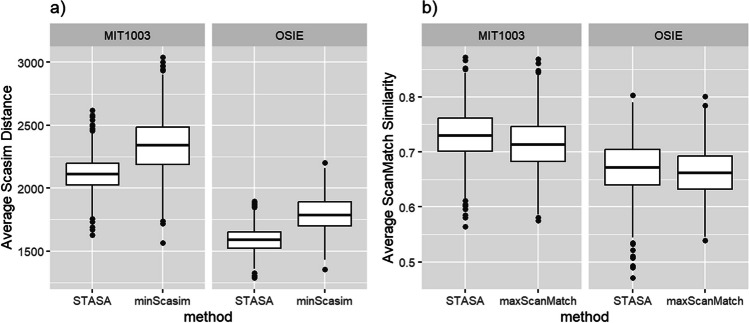
Comparison of the performance of STASA and minScasim, as well as STASA and maxScanMatch on data sets MIT1003 and OSIE

Figure 9a shows that when using Scasim as the dissimilarity measure, the representativeness of the scanpath obtained by STASA is significantly higher than that of the original scanpath in the sample for both data sets (*p* value < 0.01 by *t* test and Cohen’s *d* = 1.21 by MIT1003; *p* value < 0.01 by *t* test and Cohen’s *d* = 1.71 by OSIE). However, when using ScanMatch as the similarity measure, as shown in Fig. 9b, the representativeness of the scanpath obtained by STASA is only slightly better than the scanpath in the sample (*p* value < 0.01 from *t* test and Cohen’s *d* = 0.35 by MIT1003; *p* value = 0.01 from *t* test and Cohen’s *d* = 0.14 by OSIE). One reason for this discrepancy is that both Scasim and ScanMatch calculate the similarity of scanpath by first matching the fixations or grids and then comparing the differences between the paired fixations or grids. However, Scasim directly compares the exact position (pixel coordinates) of the fixations, while ScanMatch compares the grids where the fixations are located. Consequently, ScanMatch cannot discern significant differences between the representative scanpath generated by STASA and the representative scanpath identified by maxScanMatch, when the ScanMatch’s spatial grid is too coarse-grained.

**Fig. 10:**
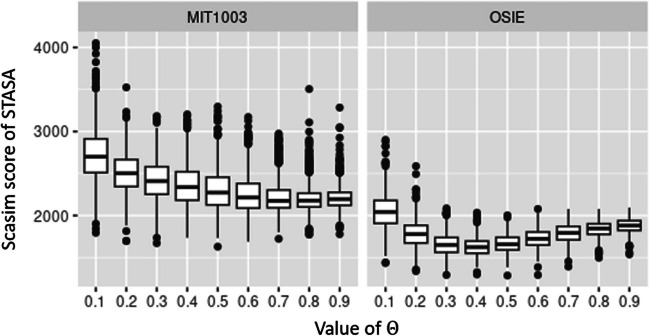
Comparison of the performance of STASA and minScasim, as well as STASA and maxScanMatch on data sets MIT1003 and OSIE

In addition, we only tried nine different theta values equally spaced between 0.1 and 0.9. Theoretically, if more values of *θ* are tried, it should be possible to generate a scanpath with higher representativeness. However, as shown in Fig. 10, we do not think it is necessary because the difference of the scanpaths obtained by the neighboring *θ*’s values on this two data sets is not significant.

## Summary and discussion

This paper introduces a new method to identify a representative scanpath in a sample by following the principle of Gestalt theory that fixations are from whole AOIs instead of single gaze points. We introduced the concept of short-time AOIs, which are the AOIs formed by the fixations on scanpaths in a short period of time and obtain the fixations of the representative scanpath from the short-time AOIs. Older methods employ global AOIs, which are formed by all the fixations on the scanpaths without considering their order and duration information.

We compared our method with the existing global AOIs-based method on two large public accessible eye-tracking data sets. The results show that the representative scanpaths found by our method have higher average similarity (representativeness) to the original scanpaths as measured by ScanMatch (Cristino et al., 2010). We also use MultiMatch (Jarodzka et al., 2010) to measure the representativeness in different aspects. Results show that the scanpaths found by our method have higher representativeness in the overall saccade vector. The representativeness of the saccade vectors is essentially determined by the position of the fixations at their two ends. This is empirical evidence of the advantages of short-time AOIs. However, the limitation of the above comparison is that since Li and Chen (2018) did not publish the code and data for the global AOI-based methods, we can only compare the average performance but without statistical tests to support the above conclusions.

Instead of generating a non-existent scanpath, an existing scanpath from the original sample can be found as a representative scanpath. This kind of method greatly reduces the computation time. We also compared our method with such simple methods. We performed the computations on a personal computer. For the two datasets used in this paper, the former can complete the computation for a single stimulus in less than a second, and STASA takes about 5 s on average. Although computation time is a disadvantage for STASA, times in the range of seconds are acceptable for general application. Regarding the representativeness, the scanpaths generated by STASA have higher representativeness than that of any existing scanpath. The above comparison depends on which scanpath similarity measure is used. STASA has a larger advantage when using Scasim as the similarity measure, which is sensitive to the location of the fixations. When using ScanMatch as the similarity measure, the advantage of STASA is no longer obvious. This is because ScanMatch represents fixations with similar locations in the same grid. If the grids are large, the differences between scanpaths will tend to be reduced. In the above comparison by ScanMatch, we partition all the stimuli into 12 x 8 grids, the same as the authors of ScanMatch did in their original paper. We expect that on finer grids, the advantage of STASA will be more obvious.

The aim of this paper is to develop a general methodology for generating representative scanpaths for a sample of scanpaths. We use Scasim to evaluate the generated scanpath and we propose a tuning strategy for the parameter of STASA in cases where the user does not specify a scanpath similarity in advance. However, potential subjectivity could arise from the user by specifying the similarity measure. This is hard to avoid because the similarity of a scanpath covers many aspects, including temporal, spatial, or semantic. Therefore, there exists no universally unbiased similarity for scanpaths, so that it is hard to generate a universally unbiased representative scanpath. However, if the user knows exactly which scanpath similarity is best suited for their specific task, our method can be adapted to that scanpath similarity by adjusting the parameter *θ*.

The introduced method in this paper is neither a scanpath clustering nor a scanpath classification method. Scanpath classification models can be used to characterize visual strategies of groups such as experts and novices, or to assign class labels, such as expertise level, to viewers depending on their scanpaths (e.g., Fuhl et al. 2019; Coutrot et al. 2018). Scanpath clustering models divide the sample into groups based on the similarity of the scanpaths with each other (e.g., Burch et al. (2018) and Haass et al. (2016)). The common task of all these methods including ours is to find view patterns for a sample of viewers. The difference is that our method is only for one group of viewers, whereas scanpath classification looks for view patterns for each of multiple groups, and scanpath clustering is to group viewers of scanpaths. Theoretically, our approach can also be used in the task of scanpath classification. For example, we can compute a representative scanpath for each class as the view pattern. A viewer can be assigned to the class who’s representative scanpath has the highest similarity to the viewer‘s scanpath. Our approach can also be a building block for scanpath clustering. A representative scanpath serves as a cluster average. For example, in the studies of von der Malsburg and Vasishth (2013), Parshina et al. (2022), and Paape et al. (2022), scanpaths are clustered into groups firstly according to their similarities with each other. Then a prototypical view pattern for each cluster is identified using an existing scanpath in the cluster, which maximizes the similarities to all other cluster members. If the scanpath generated by our method has greater average similarity to all cluster members, then it is theoretically more suitable to be the searched prototypical view pattern. However, after clustering, the scanpaths within each cluster may be highly similar to each other. It depends on which cluster method is used; some cluster methods remove outlier scanpaths while clustering, such as DBSCAN, while others do not, such as *k*-means. If there are no outliers, the scanpaths within a group are highly similar to each other, so that an existing scanpath can already be highly representative. Therefore, it is to be expected that the scanpaths generated by our method will not improve the representativeness that much. Nevertheless, in some application scenarios, scanpaths are not grouped by their similarity, but based on the subject groups (e.g., novices and experts), or experimental settings, such as the task assigned to a subject. In such situations, since our method removes the outlier fixations when constructing the short times AOIs, it can be expected to generate more representative scanpath than any original scanpath. Also, if scanpaths are noisy for short periods (e.g., realistic driving simulation), the representative scanpaths obtained by our method might have the added benefit to smooth out noise.

**Fig. 11:**
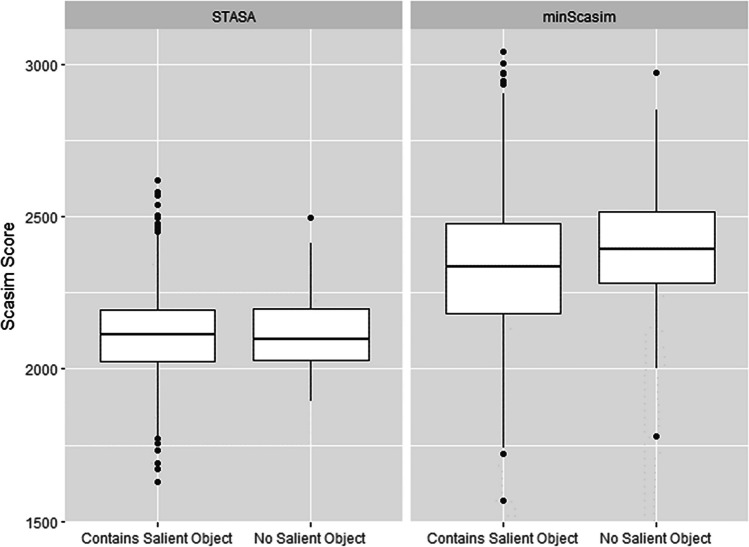
Comparison of the representativeness of the scanpaths for stimuli with and without salient objects

**Fig. 12:**
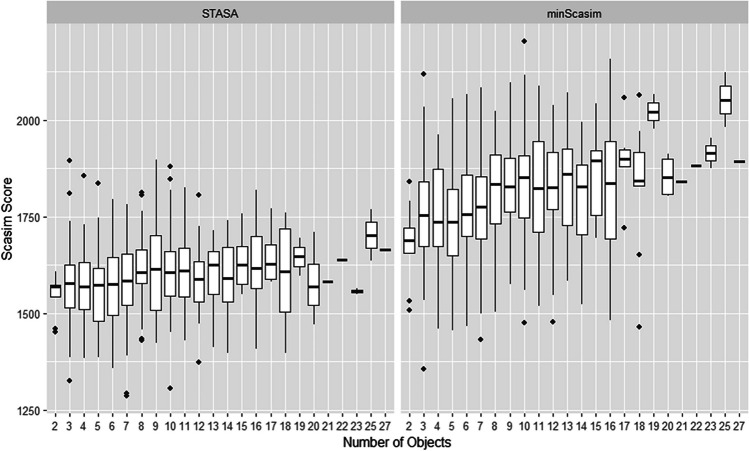
The representativeness of the representative scanpaths for stimuli with different number of objects

The noise level of a set of scanpaths also depends on the number of objects on the stimulus. One reviewer of our paper suggested a potential relationship between the stimuli and the representativeness score of the representative scanpath. That is, one could expect a higher representative scanpath for stimuli containing salient objects driving observers’ gaze and lower for stimuli of landscape scenes. We used a pre-trained deep learning salient object detection model (see Hou et al. 2017) to identify the salient objects on each stimulus of the MIT1003 data set and compared the representativeness of the scanpaths found by our method (STASA) with the scanpath minimizing the average Scasim distance (minScasim). However, as shown in Fig. 11, we did not find a noticeable difference in either of the methods. A possible reason for this is that there are too many objects in some stimuli. If only a few salient objects exist in the image, the scores of the representative scanpath should also tend to be higher on average, because if there are more salient objects in the stimuli, the observation strategies of different observers may vary widely, which leads to a lower degree of representation of the obtained representative scanpaths. We therefore used the deep learning tool YOLO3 (see Redmon and Farhadi, 2018) to automatically detect the number of objects contained in each image. As shown in Fig. 12, it is true that the score of representative scanpaths is highest (the average Scacim score is lowest) when the number of objects is only two. We can also see a slight upward trend when the number of objects increases.

## Data Availability

Two publicly available data sets (MIT1003 and OSIE) are used in this paper. They can be found for example under http://saliency.mit.edu/datasets.html.

## Code availability

The code for this paper is publicly accessible.

Our algorithm is implemented in R (R Core Team, 2021) and developed as an R package, which can be found under https://github.com/HeHuangDortmund/scanpathAggr

Code to produce the results in this paper can be found under https://github.com/HeHuangDortmund/STASA_ms
